# Effects of Paraspinal Intramuscular Injection of Atelocollagen in Patients with Chronic Low Back Pain: A Retrospective Observational Study

**DOI:** 10.3390/jcm13092607

**Published:** 2024-04-29

**Authors:** Tae Kwang Kim, Ho Young Gil

**Affiliations:** 1 Department of Anesthesiology and Pain Medicine, Ajou University School of Medicine, Suwon 16499, Republic of Korea; tk.kim@aumc.ac.kr; 2 Department of Anesthesiology and Pain Medicine, Soonchunhyang University Gumi Hospital, Soonchunhyang University College of Medicine, Gumi 39371, Republic of Korea

**Keywords:** atelocollagen, back muscles, back pain, injections, epidural, low back pain, paraspinal muscles

## Abstract

**Background/Objectives**: Atelocollagen is used for soft tissue repair and reconstruction by replacing defective or damaged muscles, membranes, ligaments, and tendons. This study aimed to evaluate the clinical efficacy and safety of additional paraspinal intramuscular injection of atelocollagen on lumbar epidural steroid injection for reducing pain and improving functional capacity of patients with chronic low back pain (CLBP). **Methods:** We retrospectively enrolled 608 consecutive patients with CLBP who received lumbar epidural steroid injection with or without additional paraspinal intramuscular injection of atelocollagen. The Numerical Rating Scale and the Oswestry Disability Index were used to assess pain and functional capacity, respectively, before the procedure, and three months after the injection. Also, we analyzed the relationship between the additional paraspinal intramuscular injection of atelocollagen and the success rate. **Results:** Both Numerical Rating Scale and the Oswestry Disability Index scores were significantly reduced in both groups at three months after injection. However, there was a significant difference between the two groups. Furthermore, the success rate was significantly higher in the additional paraspinal intramuscular injection of atelocollagen group. **Conclusions:** This study’s results showed that additional paraspinal intramuscular injection of atelocollagen on lumbar epidural steroid injection reduced pain and improved functional capacity for patients with CLBP. Therefore, the paraspinal intramuscular injection of atelocollagen may be a promising option for the treatment of patients with CLBP.

## 1. Introduction

Low back pain (LBP) is very common; it affects about 70% of people at some point in their lives [1]. It is now a leading cause of disability worldwide, which places a huge economic burden on the healthcare system [2,3]. Most (90~95%) cases of LBP are non-specific LBP, defined as LBP not attributable to a known anatomical cause [3,4]. Lumbar disc herniation is one of the most frequent causes of LBP and sciatica in adults, having a prevalence of about 1% to 3% [1]. Compression by the protruding disc on the dorsal and/or ventral nerve roots can cause LBP, leg pain, muscle spasm, and restriction of trunk movement [5].

Management for LBP includes medication (such as non-steroidal anti-inflammatory drugs, muscle relaxants, and analgesics), multidisciplinary rehabilitation, cognitive behavioral therapy, exercise, spinal manipulation, and epidural steroid injection [1,3,6]. In LBP patients, lumbar epidural steroid injection can provide short-term pain relief, enabling return to daily life and early rehabilitation [6,7,8]. It is very important in the treatment of LBP. If pain persists, physical activity will decrease which can lead to weight-gain and changes in body composition, including an increase of body fat percentage [9].

In rehabilitation, most studies and guidelines recommend exercise for LBP patients [10]. To prevent LBP from becoming chronic, exercise of paraspinal muscles (multifidus, the erector spinae, the psoas, and the quadratus lumborum) is important [11,12]. Although, there is considerable heterogeneity among studies in the type of exercise program (aquatic exercises, stretching, back schools, McKenzie exercise approach, yoga, and tai-chi) and mode of delivery (individually designed programs, supervised home exercise, and group exercise), exercises can decrease recovery time compared with no treatment [1,3].

However, it is difficult for LBP patients with impairments of paraspinal muscles to exercise, even for younger adults. They are more likely to develop CLBP [11]. As many as 33% of people still have moderate-intensity pain and 15% have severe pain at one year after an initial LBP [1]. This tendency is more pronounced in elderly patients, especially in those with sarcopenia who show loss of muscle size and an increase in fat infiltration in lower lumbar levels [11,13]. In these patients, oral supplementation with collagen can help improve body composition and strengthen muscles [14,15,16]. Also, it has a salutary effect on bone and cartilage metabolism in patients with osteoarthritis and osteoporosis [17].

Atelocollagen is a pepsin-treated Type I collagen lacking telopeptides at N and C terminals that confer antigenicity [18]. Highly purified atelocollagen has many advantages due to its biocompatibilities and ability to optimize collagen–cell interactions for efficacy while lowering side effects [19]. These properties allow the use of atelocollagen in protein and gene drug delivery systems for cancer patients [20,21]. As for therapeutic purpose of atelocollagen, it has been used to enhance wound healing [22]. Injection of atelocollagen is an effective treatment for rotator cuff tendon tear, plantar fasciitis, and osteoarthritis of the knee [19,23,24,25]. Atelocollagen-mediated myostatin-siRNA and muscle-specific miRNA can also be used to increase skeletal muscle mass and recovery of muscle function [26,27,28]. Cross-linked atelocollagen sponges have the potential as scaffolds for muscle tissue regeneration in a rabbit study [29]. In Korea, atelocollagen is approved for the purpose of soft tissue repair and reconstruction by replacing defective or damaged muscles, membranes, ligaments, and tendons.

We speculate that paraspinal intramuscular injection of atelocollagen could improve muscle mass composition and increase functional status. Therefore, it will be helpful for early recovery of patients with CLBP and increase the treatment effect of lumbar epidural steroid injection. Thus, the purpose of this study was to assess the efficacy and safety of additional paraspinal intramuscular injections of atelocollagen on lumbar epidural steroid injection for reducing pain and improving the functional capacity of patients with CLBP.

## 2. Materials and Methods

This retrospective observational study was approved by the Institutional Review Board (IRB) of Ajou University Hospital of Korea (IRB No. AJOUIRB-MDB-2022-242) in July 2022. The requirement for informed consent was waived because of the retrospective case–control nature of this study.

### 2.1. Participants

**Figure 1:**
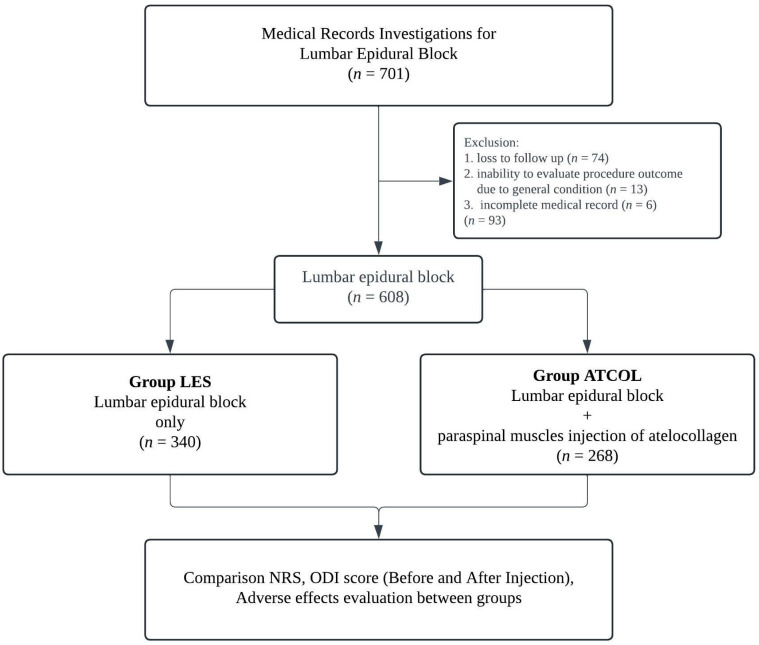
A flow diagram of research.

From March 2018 to April 2022, we retrospectively enrolled 701 consecutive patients with CLBP who received lumbar epidural steroid injections with or without additional paraspinal intramuscular injection of atelocollagen. The diagnosis was determined after assessing the patient’s symptoms, neurological examination results, and imaging studies. The inclusion criteria were as follows: (1) age between 20 and 80 years old, (2) CLBP with or without leg radicular pain (NRS ≥ 4), (3) lack of responses to conservative therapy for more than three months, and (4) epidural steroid injection with or without additional paraspinal intramuscular injection of atelocollagen. The exclusion criteria were: (1) loss to follow-up, (2) inability to evaluate procedure outcome because of other severe diseases such as cancer, and (3) incomplete medical records. Patients were regularly followed up until three months after the procedure. Finally, 93 patients were excluded based on the criteria, and a total of 608 patients were evaluated. Patients were divided into two groups: a control group consisting of patients who received lumbar epidural steroid injection only (Group LES, *n* = 340) and a group of patients who received additional paraspinal intramuscular injection of atelocollagen on lumbar epidural steroid injection (Group ATCOL, *n* = 268) (Figure 1).

### 2.2. Lumbar Epidural Steroid Injection

A pain physician, who has over a decade of experience, performed an epidural steroid injection on an outpatient basis. Epidural steroid injection was performed via an interlaminar approach under c-arm. The patient was placed in a prone position and the area where the injection would be performed was disinfected. An anterior–posterior view was used to identify the target lumbar intervertebral space. The skin was anesthetized with 1% mepivacaine and a 20-gauge Touhy needle was advanced until encountering resistance at the ligamentum flavum while intermittently imaging. Depth was assessed using lateral imaging. When the ligamentum flavum resistance was felt, the stylet was removed, and the needle was placed in the epidural space using a loss-of-resistance technique. After penetrating the ligamentum flavum, 3 mL of a nonionic contrast media (Iopamiro 300 inj.; Bracco Imaging Korea, Ltd., Seoul, Republic of Korea) was administered to confirm epidural placement. Once optimal contrast spread was seen, 1500 units of hyaluronidase in 2 mL preservative-free normal saline, 5.5 mL of 0.3% mepivacaine and 2.5 mg (0.5 mL) of dexamethasone mixture were injected [30].

### 2.3. Atelocollagen Injection

**Figure 2:**
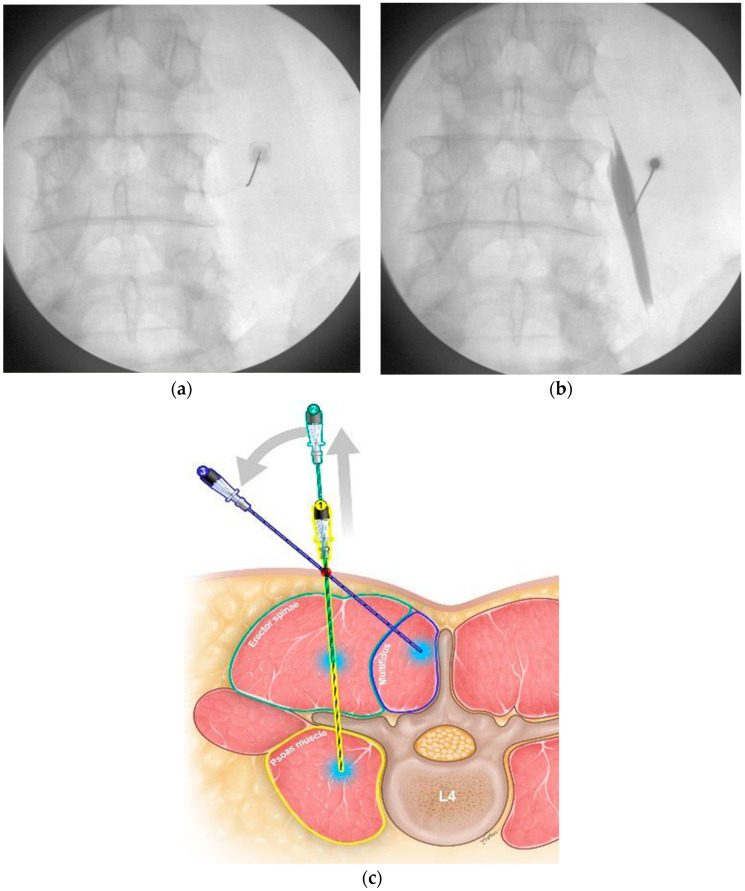
Additional injection to paraspinal muscles was consecutively performed under c-arm. (**a**) A 22-G Tuohy needle touched the transverse process. (**b**) Fluoroscopic images were taken to confirm the position of the needle in the psoas muscle. (**c**) Illustration of an injection into each paraspinal muscle. ① The yellow needle is an illustration of an injection into the psoas muscle, ② the green needle is an illustration of an injection into the erector spinae, and ③ the blue needle is an illustration of an injection into the multifidus.

Additional injection of atelocollagen to paraspinal muscles was consecutively performed under c-arm. The patient was placed in a prone position and the area where the injection would be performed was disinfected. Using an anterior–posterior view, transverse processes of the L4 were confirmed and a 22-G Tuohy needle was inserted [31]. Once the transverse process was contacted, the needle was slightly pulled back and advanced below the transverse process (Figure 2a). After intermittent injection of contrast agent, fluoroscopic images were taken to confirm the placement of the needle within the psoas muscle (Figure 2b). Then, 5 cc of atelocollagen mixture was injected. Second, erector spinae injection was performed. The needle in the psoas muscle was pulled back to the depth at which it touched the transverse process of the L4. The needle was pulled back 0.5~1 cm further from the depth where the transverse process was touched and 2.5 cc of atelocollagen mixture was injected into the erector spinae (Figure 2c) [11,32]. Third, the needle in the current position was adjusted to the medial side so that the needle could touch the laminar or spinous process. The needle was then pulled back 0.5~1 cm and 2.5 cc of atelocollagen mixture was injected into the multifidus (Figure 2c). Fluoroscopic images were taken intermittently as needed to identify the proper position of the needle in each muscle. We injected a total of 10 mL of atelocollagen mixture into one side of the lumbar spine. Usually, we mixed 1 mL of atelocollagen (CollaShield^®^, DALIM TISSEN Corp, Seoul, Republic of Korea) and 9 mL of 0.3% mepivacaine. Depending on the patient, 0.5 mL of atelocollagen or more was used.

### 2.4. Post-Injection Care

After the procedure, all patients were monitored for 30 min to check for any neurological deficits or other complications related to the procedure. Before being discharged, we gave the patients instructions on precautions to follow post-injection (such as injection-related infection, swelling, and pain) and information about core muscle strengthening exercises.

### 2.5. Evaluation of Outcome Variables

Patients assessed their symptoms by filling out questionnaires (numerical rating scale, Oswestry Disability Index score) before and three months after injection. The patient’s pain level was quantified using a standardized 11-point numerical rating scale (NRS), ranging from 0 to 10. A well-trained physician assessed this at baseline and again three months following the injection. The intensity of pain was rated on a scale from 0 to 10 (0: no pain; 10: the worst pain). Patients were encouraged to describe their feelings regarding the pain. The Oswestry Disability Index (ODI) score was utilized to evaluate the level of dysfunction in patients. ODI assessments were conducted initially and three months post-injection. The ODI is a 10-item questionnaire widely used internationally to evaluate the functional status of patients with CLBP. In this study, we utilized the 9-item Korean version of the ODI, which omits the assessment of sexual function due to cultural considerations [33]. Treatment success was defined as achieving a reduction of more than 50% in the NRS score three months following the injection. The relationship between additional paraspinal intramuscular injection of atelocollagen on lumbar epidural steroid injection and success rate was also analyzed.

### 2.6. Statistical Analysis

When estimating the sample size for a pilot study, the total sample size was 312 patients when the significance level was 0.0500 and the power was 0.8. An independent samples *t*-test was used for continuous variables and a Chi-square test was used for categorical variables. Paired samples *t*-tests and independent samples *t*-tests were used to compare NRS and ODI of the two groups. All statistical analyses were performed using R software, version 4.1.0. A *p*-value of less than 0.05 was considered to be statistically significant.

## 3. Results

**Figure 3:**
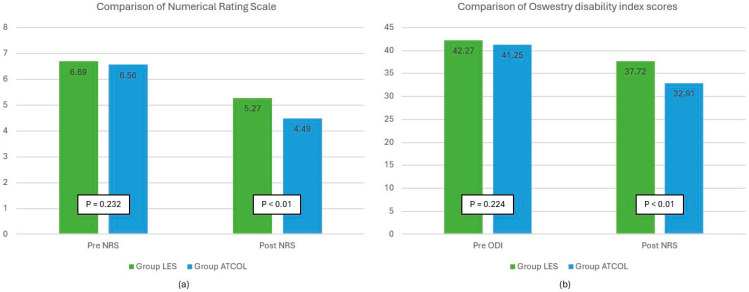
Differences in (**a**) Numerical Rating Scale and (**b**) Oswestry Disability Index scores before and after the procedure by group.

Demographic data are shown in Table 1. There were significant differences in age, gender, and height between ATCOL and LES groups. The ATCOL group had significantly more elderly and short patients. The proportion of women was also significantly higher in the ATCOL group. NRS and ODI scores are shown in Table 2 and Figure 3. Changes in NRS and ODI scores were analyzed at three months after injection compared to those before injection. Both NRS and ODI scores were significantly reduced in both groups at three months after injection. However, they were significantly different between the two groups, Table 2. Furthermore, the success rate was significantly higher in the ATCOL group than in the LES group (Table 3). Of 268 patients, 112 (41.79%) complained of symptoms on one side only and received injections unilaterally. The average volume of atelocollagen mixture injected into one side of the lumbar spine was 0.96 ± 0.66 mL, and there were no serious side effects associated with ATCOL injection.

## 4. Discussion

In this study, we found that additional paraspinal intramuscular injections of atelocollagen on lumbar epidural steroid injections (Group ATCOL) was significantly more effective than lumbar epidural steroid injection alone in reducing pain in patients with CLBP. Although changes of muscle mass and composition on MRI and muscle strength could not be evaluated, which was a shortcoming of our study, significantly improved functional capacity was observed in CLBP patients, and our results indicate that if patients can start early rehabilitation through this treatment, their muscle mass and composition will naturally improve. Based on results of this study, we propose that additional paraspinal intramuscular injection of atelocollagen on lumbar epidural steroid injection could be one of the treatment options for CLBP patients who have paraspinal muscle impairment.

### 4.1. Paraspinal Muscles in LBP Patients

Skeletal muscle is an important body-composition component of locomotion that plays a very important role in maintaining exercise balance, glucose metabolism, and energy metabolism of the body [34,35]. In particular, paraspinal muscles support and control movement of the lumbar spine. Impaired muscle support is a factor that persists LBP. Therefore, rehabilitation is very important for patients with LBP [12].

Many studies have investigated changes of paraspinal muscles in patients with LBP by measuring cross-sectional area (CSA) and fat content using CT or MRI [36,37]. Although there was conflicting evidence for a relationship between CSA and fat content of paraspinal muscles and LBP, current evidence suggests that paraspinal muscles are smaller CSA in patients with CLBP than in healthy individuals of similar ages [5,37].

Sions et al. [11] determined whether there are differences in trunk muscle characteristics between older adults with CLBP (*n* = 53) and those without CLBP (*n* = 49) in MRI while controlling for age, sex, and body mass index. Older adults with LBP had a greater fat content of multifidus and smaller average CSA of erector spinae than those in the control group without LBP. They reported that up to 54% of older adults with trunk muscle CSA might be fat. Also, women had smaller muscles and greater intramuscular fat than men. We speculate that this may be the reason why the proportion of women was significantly higher in the ATCOL group in this study.

In a systematic review, Goubert et al. found that lower CSA of the multifidus had moderate evidence in CLBP, whereas it was less clear in other paraspinal muscles. There was no decrease in CSA in those with recurrent LBP and acute LBP. Although an increase in fat content was shown in CLBP, they thought it was more likely to be due to age or by disuse of lumbar muscles rather than due to LBP itself [13].

The psoas stabilizes lumbar spine together with lumbar back muscles. The psoas index (bilateral psoas muscle CSA/height^2^) has been found to be associated with the CSA and ratio of functional CSA (subtracting the fat infiltration area from the CSA) of the multifidus and erector spinae at L4–5 and L5–S1 levels [38]. Additionally, the psoas index decreased with increasing age.

Arbanas et al. [39] studied the CSA of the psoas in LBP patients. They found that the CSA of the psoas was significantly bigger at L3/4 and L4/5 levels. They concluded that it might be a result of its increased activity because of an instability associated with degenerative disorders of the lumbar spine. They also assumed that the psoas muscle remained active regardless of degenerative changes in the lumbar spine. However, patients with apparent degenerative changes of the lumbar spine had significantly smaller CSA of the psoas at the same levels.

### 4.2. Skeletal Muscles with Aging

Aging is associated with loss of skeletal muscle mass (sarcopenia) and function (fibrosis and extracellular matrix deposition) [40,41]. It has been observed to decrease with age at a rate of approximately 1% annually after age 40 [42]. Aging and decline of physical activity can lead to progressive atrophy and loss of individual muscle fibers associated with concomitant loss of motor units [43]. In addition, there is infiltration of fat and other non-contractile materials, which can cause a reduction in muscle ‘quality’ [44].

Stearns-Reider et al. [45] quantitatively analyzed the topological structure of extracellular matrix (ECM) and mechanical properties of muscles, showing that with age, collagen bending decreases, ECM stiffness increases, and mechanical properties of skeletal muscle decreases. Age-associated muscle fibrosis characterized by a loss of clear two-directional lattice orientation of healthy perimysial collagen fibers can increase muscle stiffness [46]. Absolute collagen content and densely packed collagen might be increased. Collagen composition is also changed (shift toward higher Type I to Type III collagen). Collagen Type IV concentration is enhanced in the basal lamina of slow twitch muscles, whereas laminin concentration seems to decrease with age [35]. Extensive non-enzymatic cross-linking of collagen fibers might be increased. The elastic modulus of the ECM can be increased approximately 35-fold [47]. Age-associated changes in the ECM might be driven by a decreased degradation capacity rather than by increased synthesis of collagenous structures [35].

### 4.3. Extracellular Matrix in Muscle Generation and Repair

Muscle fibers and satellite cells are the predominant stem cell population in adult skeletal muscles. They reside in the skeletal muscle microenvironment also known as a niche [34]. ECM is a kind of connective tissue in the muscle cell microenvironment. Present in the muscle niche, it is composed of collagen Types I and III, fibronectin, laminin, glycoproteins, proteoglycans, elastin, and RNA [48,49]. ECM can regulate muscle development, growth, and repair. It is essential for effective muscle contraction and force transmission [35,50].

Collagen is the largest component of ECM protein in skeletal muscles. The collagen superfamily contains a total of 28 different members. Collagen types expressed in adult striated muscles and in muscles during development include Types I, III, IV, V, VI, VIII, XII, XIII, XIV, XV, XVIII, and XIX [35].

Collagens can form a lattice fiber network of intramuscular connective tissue (IMCT) such as central, fibrous components of the ECM. IMCT acts as a scaffold for muscle fiber development and growth and acts as a carrier for blood vessels and nerves to muscle cells [35]. The variability of IMCT between different muscles represents the role of changes in active and passive mechanical properties of muscles [51,52]. The IMCT contains various forms of collagens with fibril-forming Types I and III being the most abundant. Types I and III collagen account for approximately 75% of total muscles. They are present in all anatomical entities of muscles [53]. The IMCT can be divided into three independent and interconnected layers: (1) the endomysium, representing the innermost layer that encloses individual muscle fibers; (2) the perimysium bundling group of muscle fibers; and (3) the epimysium enveloping the entire muscle [34]. The endomysium contains predominantly Types I, III, and V, perimysium Types I and III, and epimysium Type I collagens. Type I collagen is present in the endo-, peri-, and epimysium. It characteristically forms strong parallel fibers and confers tensile strength and rigidity, whereas Type III collagen forms a loose meshwork of fibers, giving elasticity to the endo- and perimysium. Although Type V collagen is present in small amounts, it plays a fundamental role in the control of collagen fibrillogenesis [51,54,55].

In the genesis of ECM during muscle fiber development using zebrafish, an orthogonal dense network of collagen fibers is gradually formed to anchor the myoepithelium or fibroblasts to the basal lamina [56]. The basal lamina is a supramolecular ECM structure. The integrity of the basal lamina is the basis of regeneration of damaged muscle fibers [57]. The connection between the basal lamina is mainly made up of the strut of collagen I, which contains collagen fibers, elastin fibers, and microfibrils. The rest is filled with a polyanionic lattice of unit collagen fibers, microfilaments, and particles. Furthermore, the basal lamina contains a variety of growth factors that directly participate in the physiological activities of muscle fibers and play an important role in maintaining physiological functions of skeletal muscles [34].

Rapid repair of muscle damage is as important as muscle development and growth. In damaged muscles, damaged or dead fibers are first removed by inflammatory cells. Cytokines and growth factors are released from both injured blood vessels and infiltrating inflammatory cells [58]. Quiescent satellite cells beneath the basal lamina of muscle fibers become activated, followed by their extensive proliferation [35,40]. Differentiated myoblasts finally fuse into mature myofibers. Impairment of these stages can result in unsuccessful muscle regeneration, usually characterized by persistent degeneration, inflammation, and fibrosis of muscle fibers [40].

Migration and proliferation of fibroblasts to produce new temporary ECM components are also required for efficient muscle repair. ECM hyperplasia (increase in collagen concentration) can result in increased skeletal muscle tissue stiffness. This orderly deadhesion, and fibrosis is designed to protect skeletal muscle from further damage [34]. ECM can improve muscle function to a certain extent by regulating force transmission at the injured site rather than by relying on skeletal muscle regeneration [59].

Genes associated with ECM remodeling having an important role in skeletal muscle regeneration are up-regulated. Activation of satellite cells can induce local remodeling of ECM to repair the damaged basal lamina. ECM can release cytokines to promote proliferation of myogenic progenitor cells and regeneration of myofiber [34,60]. Temporary ECM components are also crucial for guiding the formation of neuromuscular junctions [61]. The ECM environment can regulate synaptogenesis in the process of synaptic induction. Integrity of the neuromuscular junction and transduction of synaptic signals are keys to motor function of skeletal muscles [34,62]. ECM proteins could promote the activity of acetylcholinesterase [63,64].

### 4.4. Clinical Use of Collagen

Collagen is attractive as a potential therapeutic agent in osteoarthritis and osteoporosis patients [17,65]. Moskowitz [17] studied 52 patients with degenerative hip or knee disease. Patients were taken daily with 10 g of tablet form of collagen orally for four 60-day with a 2-month washout between each treatment. German patients showed a statistically significant improvement in pain relief. In addition, increased efficacy for collagen supplements was observed in the overall study population amongst patients with more severe symptomatology at study onset. Preferential uptake of radiolabeled proline in collagen hydrolysate, as compared to labeled free proline, suggests that collagen supplements may play a positive role in cartilage metabolism. There were no serious side effects associated with collagen supplementation. Most side events were mild to moderate gastrointestinal disorders. Also, in 121 osteoporosis patients, patients treated with a combination of calcitonin and collagen supplementations had a significantly greater fall in urinary cross-links as compared with patients treated with calcitonin alone. They suggested that a combination of calcitonin and collagen supplementations had a greater effect in inhibiting bone collagen breakdown.

Also, injection of atelocollagen appears to be an effective method for cartilage regeneration. In a rabbit study, injection of a mixture of atelocollagen and fibrin was used to treat articular cartilage defects of the knee. The surface of the newly generated cartilage was very smooth and even. It was also noted that the entire area was completely regenerated [66]. A study was also conducted on intra-articular injection of atelocollagen in 200 patients with osteoarthritis, chondromalacia, or other cartilage defects. The degree of pain was significantly reduced in patients who received intra-articular injection of atelocollagen [25].

The muscle mass and strength can be further increased through collagen supplementation combined with resistance exercise in elderly subjects [14]. A total of 53 male participants with sarcopenia underwent a 12-week guided resistance training program. They were supplemented with either collagen peptides (15 mg/d) or silica as placebo. In both groups, fat-free mass was significantly increased whereas fat mass was significantly decreased. Muscle strength, sensory motor control, and bone mass were significantly improved. However, the effects were significantly more pronounced in subjects receiving collagen supplementations. Collagen is rich in arginine and glycine, which are known to be important substrates for creatine synthesis in the human body. Creatine supplementation has been shown to be able to improve muscle mass and function in some studies and reduce sarcopenia [67,68]. Also, collagen has been shown to have a positive effect on microcirculation, which may lead to additional beneficial effects in promoting muscle growth [69].

Collagen is used for tendon treatment. In rotator cuff tear, collagen sponges and patches have been used for augmentation [70,71]. Tenocytes are embedded in an extensive three-dimensional network of ECM components consisting predominantly of collagen Type I fibrils (>95% of tendon collagen). They are responsible for the synthesis and maintenance of a mechanically unique connective tissue capable of withstanding the high tensile forces subjected to tendon in vivo [72]. Injection of atelocollagen can improve functional outcome and integrity of the tendon in intra-tendinous partial-thickness supraspinatus tears [23]. Intra-tendinous injection with 0.5 mL or 1 mL of atelocollagen could significantly improve functional and pain scores (mean follow-up period was 24.7 months). Moreover, the proportion of patients with a decrease in size of the torn tendon on follow-up MRI at least 6 months after atelocollagen injection was significantly higher in the atelocollagen injection group. In histological and biomechanical studies, the injection of atelocollagen significantly enhanced the repair of rabbit supraspinatus tendon [19].

Plantar fasciitis is also an indication for injection of atelocollagen. The plantar fascia is a fibrous sheet composed primarily of Type I collagen, similar to tendons. In one study, 16 patients with plantar fasciitis were injected atelocollagen in the heel [24]. Each plantar fascia was then examined by gray-scale scanning and US elastography. The mean strain ratio of the plantar fascia was significantly increased, indicating an increased elasticity of the fascia after collagen injection. Softening of the plantar fascia decreased significantly after injection, indicating hardening (healing) of the plantar fascia after collagen injection treatment.

Cross-linked atelocollagen sponges have the potential to serve as scaffolds for muscle tissue regeneration in a rabbit study [29]. In 18 male rabbits, a muscle defect (1.0 * ~1.0 * ~0.5 cm) was created in the vastus lateralis of both legs. A piece of cross-linked atelocollagen sponge was then inserted into the defect in one leg. At 4 weeks, the sponge–muscle junction was soft and similar in color to the nearby normal muscle. However, the surface was slightly concave. No scar, contracture, or adhesion to the fascia was observed even after four weeks. The collagen sponge remained in place between muscle stumps for two weeks. It induced platelet aggregation around them, limiting hematoma development and inflammatory cells infiltration. These might prevent the excessive development of scar tissue and allow elongation of regenerating myofibers into the center of the defect. However, in the control, the defect was replaced by firm scar tissue and severe shrinkage was evident.

Our study has several strengths. First, this is the first trial examining the effect of paraspinal intramuscular injection of atelocollagen. This could be used as the basis for new attempts and studies using atelocollagen. Second, paraspinal intramuscular injection of atelocollagen could enable early rehabilitation. LBP guidelines regularly recommend the use of physical exercise. The aim of physical exercise is to improve pain and function and prevent disability from getting worse [10]. Third, paraspinal intramuscular injection of atelocollagen has the advantages of being less time-consuming, less expensive, and easier to perform than other spinal procedures or surgery.

This study also has several limitations. First, this study was retrospective in nature. Second, an ultrasound was not used for paraspinal intramuscular injection of atelocollagen. Accurate paraspinal intramuscular injection using ultrasound is required and additional research on appropriate dose of each paraspinal intramuscular injection is needed in the future. Third, we could not analyze differences in histological and radiological methods. Fourth, this study population was not homogeneous in terms of age, sex, or height. Because atelocollagen is not covered by insurance, relatively vulnerable patients received injection of atelocollagen. Considering this, it could be interpreted that there might be more significant differences between the two groups. Fifth, this study did not exclude the effects of some medications (NSAIDs, acetaminophen, and muscle relaxant, etc.) or other conservative treatments (type of exercise program and mode of delivery). Due to the limitations of this study, better designed studies are required in the future.

## 5. Conclusions

The results of this study showed that paraspinal intramuscular injection of atelocollagen on lumbar epidural steroid injection could reduce pain and improve functional capacity for patients with CLBP. Therefore, paraspinal intramuscular injection of atelocollagen might be a promising option for treating patients with CLBP.

## Figures and Tables

**Table 1:** Demographic characteristics of patients in each group.

Variable	Group		*p*-Value
	Group LES (*n* = 340)	Group ATCOL (*n* = 268)	
Age			<0.001 *
Mean (SD)	58.55 (15.74)	69.69 (13.06)	
Gender			<0.001 *
1 (male)	154 (45.3%)	79 (29.5%)	
2 (female)	186 (54.7%)	189 (70.5%)	
Height			<0.001 *
Mean (SD)	162.29 (9.60)	158.60 (9.05)	
Weight			0.019
Mean (SD)	63.96 (12.89)	61.69 (10.89)	

Data are presented as “Mean (SD) “ and “Median (Q1, Q3) for continuous variables and “*n* (%)” for categorical variables. Independent samples *t*-test was used for continuous variables and Chi-square test were used for categorical variables. SD = standard deviation. * *p* < 0.05.

**Table 2:** Comparison of numerical rating scale and Oswestry Disability Index scores before and 3 months after injection.

Variable	Pre NRS	Post NRS	NRS Difference	† *p*-Value
Group LES	6.69 (1.33)	5.27 (1.77)	1.41 (1.58)	<0.001
Group ATCOL	6.56 (1.21)	4.49 (1.85)	2.07 (1.68)	<0.001
‡ *p*-value	0.232	<0.001	<0.001	
	**Pre ODI**	**Post ODI**	**ODI Difference**	**† *p*-Value**
Group LES	42.27 (11.05)	37.72 (12.83)	4.55 (6.39)	<0.001
Group ATCOL	41.25 (9.60)	32.91 (12.43)	8.34 (7.90)	<0.001
‡ *p*-value	0.224	<0.001	<0.001	

† *p*-values were obtained using paired samples *t*-test. ‡ *p*-values were obtained using independent samples *t*-test. *p* < 0.05.

**Table 3:** Comparison of success rate between Group LES and Group ATCOL.

Variable	Group LES	Group ATCOL	*p*-Value
Successful/Total	54/340 (15.9%)	84/268 (31.3%)	<0.001 *

Chi-square test was used. * *p* < 0.05.

## Data Availability

The datasets used and/or analyzed during this study are available from the corresponding author upon reasonable request.
